# Superelastic and pH-Responsive Degradable Dendrimer Cryogels Prepared by Cryo-aza-Michael Addition Reaction

**DOI:** 10.1038/s41598-018-25456-y

**Published:** 2018-05-08

**Authors:** Juan Wang, Hu Yang

**Affiliations:** 1 Department of Chemical and Life Science Engineering, Virginia Commonwealth University, Richmond, Virginia 23219 United States; 2 Department of Pharmaceutics, Virginia Commonwealth University, Richmond, Virginia 23298 United States; 3 Massey Cancer Center, Virginia Commonwealth University, Richmond, Virginia 23298 United States

## Abstract

Dendrimers exhibit super atomistic features by virtue of their well-defined discrete quantized nanoscale structures. Here, we show that hyperbranched amine-terminated polyamidoamine (PAMAM) dendrimer G4.0 reacts with linear polyethylene glycol (PEG) diacrylate (575 g/mol) via the aza-Michael addition reaction at a subzero temperature (−20 °C), namely cryo-aza-Michael addition, to form a macroporous superelastic network, i.e., dendrimer cryogel. Dendrimer cryogels exhibit biologically relevant Young’s modulus, high compression elasticity and super resilience at ambient temperature. Furthermore, the dendrimer cryogels exhibit excellent rebound performance and do not show significant stress relaxation under cyclic deformation over a wide temperature range (−80 to 100 °C). The obtained dendrimer cryogels are stable at acidic pH but degrade quickly at physiological pH through self-triggered degradation. Taken together, dendrimer cryogels represent a new class of scaffolds with properties suitable for biomedical applications.

## Introduction

The Michael addition is a versatile synthetic method conjugating electrophilic olefins to nucleophiles typically in the presence of a base catalyst. It has been applied to synthesize various polymer architectures including linear, branched and network polymers^1–3^. The Michael addition has also been successfully applied to functionalize biologically active polymers to generate bioconjugates for biomedical and pharmaceutical applications^4^. The Michael addition involving nitrogen electrophiles as Michael donors is commonly referred to as the aza-Michael addition. Additional base may be unnecessary since amine-containing Michael donors can serve as base in the reaction. However, it is not uncommon that the aza-Michael addition has to take place in harsh organic solvents and at high temperatures to meet reaction efficiency. We recently reported that following the aza-Michael addition, the nucleophilic amines on the hyperbranched polyamidoamine (PAMAM) dendrimer surface react with *α*, *β*-unsaturated ester of the terminal acrylate groups in linear polyethylene glycol diacrylate (PEG DA) in water to form a cross-linked network^5^. This dendrimer-PEG DA aza-Michael addition is a green approach as it proceeds efficiently at room temperature without the use of a catalyst. A broad range of physical properties of the resulting dendrimer hydrogels can be fine-tuned including solidification time, rheological behavior, network structure, swelling, and degradation. Dendrimers exhibit super atomistic features by virtue of their well-defined discrete quantized nanoscale structures^6,7^. Using dendritic super atomistic building blocks to make higher order structures such as dendrimer hydrogels has expanded the utility of dendrimers in biomedical applications^8–10^.

In this work, we report that dendrimer cryogels can be prepared using the aza-Michael addition at subzero temperatures, namely cryo-aza-Michael addition. Cryopolymerization uses ice crystals as porogens to obtain macroporous materials^11–22^. There is a tremendous interest in developing macroporous monolithic elastic networks for various biomedical and tissue engineering applications^23–31^. We show that EDA-core PAMAM dendrimer G4 reacts with PEG DA via the cryo-aza-Michael addition to form a macroporous superelastic network, i.e., dendrimer cryogel. Dendrimer cryogels display distinctly different structure and properties including stiffness, flexibility, and resilience from dendrimer hydrogels prepared via the aza-Michael addition at room temperature. To the best of our knowledge, cryo-aza-Michael addition is a new method to prepare elastic dendritic networks. This method is simple and environmentally friendly. It can be employed to produce materials with tunable mechanical properties. The resulting dendrimer cryogels are highly macroporous, and they exhibit superelasticity, biologically relevant Young’s modulus, and high resilience over a broad temperature range and retains such properties following storage at various temperatures. Furthermore, the dendrimer cryogels show pH-dependent swelling and self-triggered degradation behaviors.

## Results and Discussion

### Characterization

**Figure 1:**
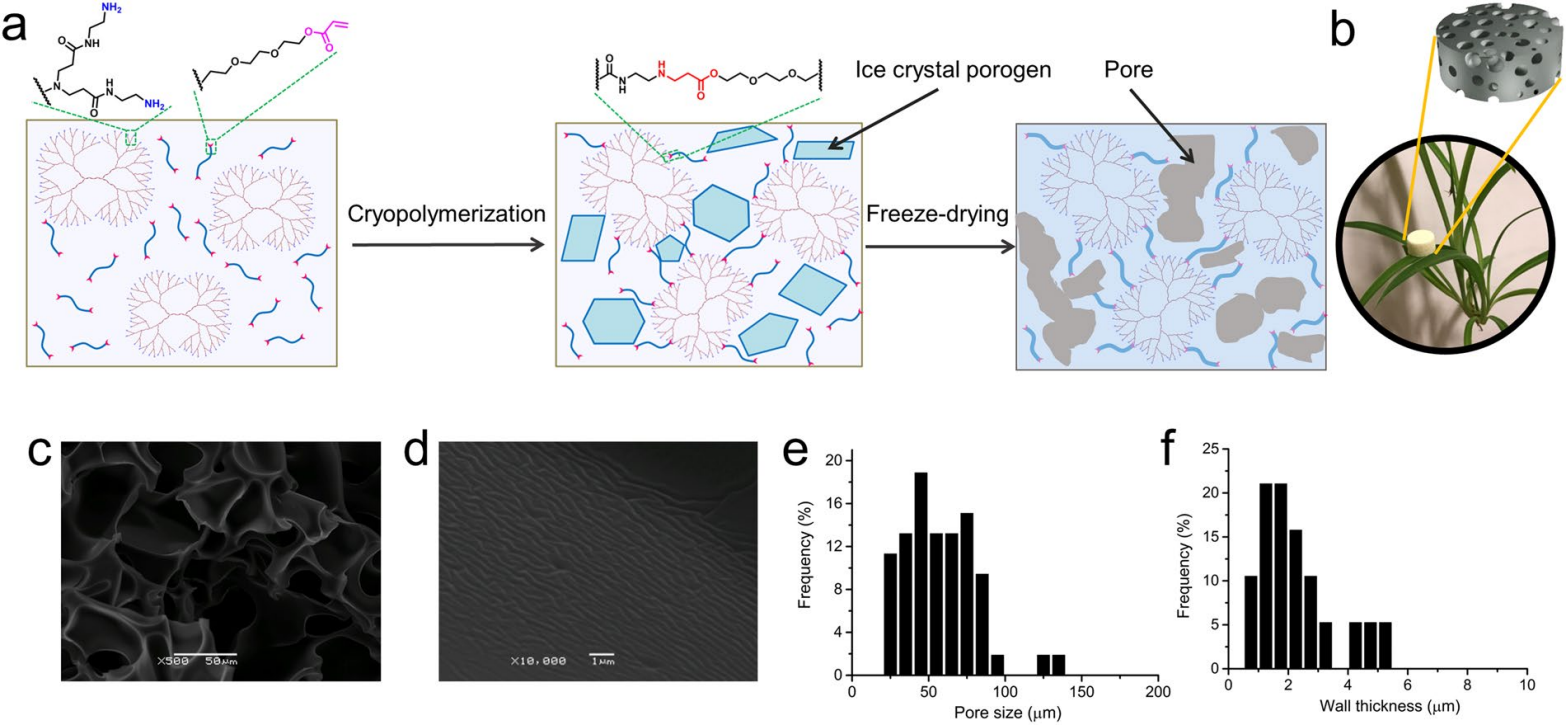
Fabrication, morphological illustration, and characterization of dendrimer cryogel CG-G4-5%. (**a**) Schematic illustration of the preparation of the dendrimer cryogel by aza-Michael addition-based cryopolymerization and freeze-drying. (**b**) A leaf supports the elastic dendrimer cryogel, suggesting its ultralight weight (density: 0.27 g/cm^3^). (**c**) SEM image reveals its porous structure. (**d**) SEM image inset shows the anisotropic surface texture of the pore wall. (**e**) Pore size distribution in the dendrimer cryogel. (**f**) Pore wall thickness distribution in the dendrimer cryogel.

Ethylenediamine core PAMAM dendrimer G4 and PEG diacrylate (575 g/mol) were mixed at the equimolar ratio of amine to acrylate in water. The mixture was incubated at a subzero temperature (−20 °C) overnight for cryopolymerization via the aza-Michael addition reaction (Fig. 1a). Ice crystal porogens form during the cryopolymerization. Upon the completion of the reaction, the ice crystals are removed by freeze-drying and leave behind interconnected pores. The dendrimer cryogel formed at 5 wt% (CG-G4-5%) is monolithic and ultralight (0.27 g/cm^3^) (Fig. 1b). The SEM images (Fig. 1c) reveal an interconnected micron-sized porous structure, which is attributed to the sublimation of ice crystals during the freeze-drying procedure. The inset amplifies the pore wall and discloses its anisotropic surface texture (Fig. 1d). The pore size of the dendrimer cryogel ranges from 25 to 135 μm, and the mean pore size is 60 ± 17 μm (Fig. 1e). The pore wall thickness range is 0.5–5 μm, and the average is 2.3 ± 1.3 μm (Fig. 1f). The dendrimer cryogels formed at 1 wt% and 10 wt% are also macroporous (Figure S1) but have different morphological structures and macroscopic appearances. CG-G4-1% is soft and fluffy (Figure S1a). It is because the concentration of dendrimer is too low to form a 3-dimensional scaffold. The morphology of CG-G4-1% looks like a piece of wrinkled paper, and its pore spatial distribution is random (Figure S1b). CG-G4-10% is light (Figure S1c) and exhibits a continuous porous network with the pore dimension smaller than that of CG-G4-5% (Figure S1d). The densities for CG-G4-1% and CG-G4-10% are 0.08 g/cm^3^, and 0.58 g/cm^3^, respectively.

Using hexane uptake (a non-solvent commonly used to calculate the pore volume of hydrophilic gels) the porosities of CG-G4-1%, CG-G4-5%, and CG-G4-10% are 89.6 ± 8.8%, 68.8 ± 6.8%, and 61.7 ± 1.2%, respectively (Figure S2). The porosity of CG-G4-1% is the highest, followed by CG-G4-5% and CG-G4-10%, which is in agreement with the SEM morphologies. We applied FTIR to qualitatively monitor acrylate groups and primary groups in the cryogels. The N–H bending vibration of primary amines is observed at 1560 cm^−1^. The C=C stretching vibration of vinyl group in PEG DA has a peak at 1720 cm^−1^. Acrylate is fully used in the reaction as the C=C stretching peaks in all the three cryogels have vanished almost completely. However, the N–H bending peaks are still noticeable in the cryogels (Figure S3), indicating secondary amines may have participated in the aza-Michael addition reaction with PEG DA.

### pH-Dependent Swelling and Degradation

**Figure 2:**
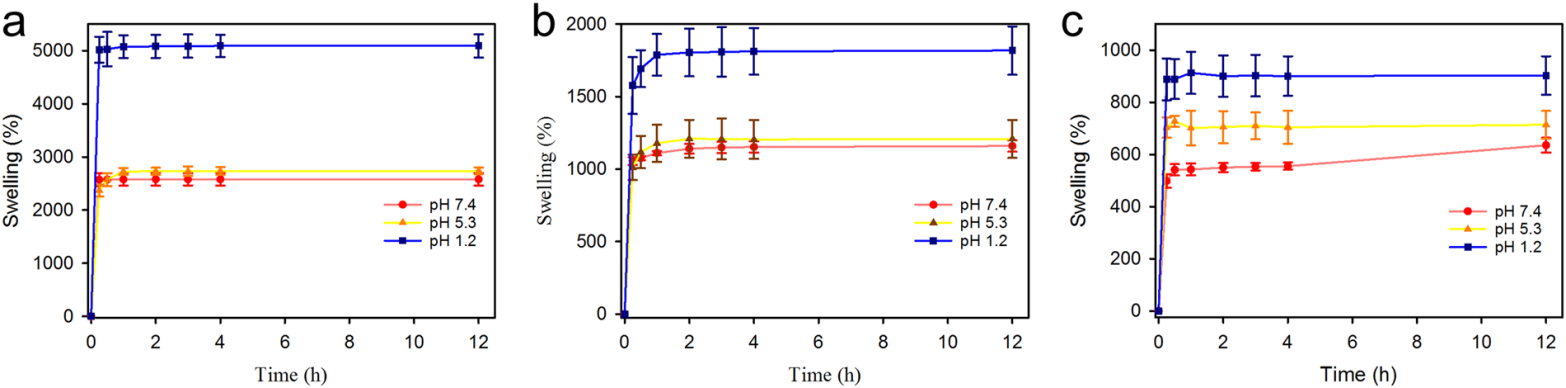
Swelling behaviors of dendrimer cryogels at 37 °C. (**a**) Effect of pH on the swelling of CG-G4-1%. (**b**) Effect of pH on the swelling of CG-G4-5%. (**c**) Effect of pH on the swelling of CG-G4-10%.

The cryogel swelling behaviors at 37 °C were examined at pH 7.4, pH 5.3, and pH 1.2. All the three cryogels, CG-G4-1%, CG-G4-5% and CG-G4-10%, show a rapid swelling rate in the first 1 h and reach equilibrium within 1 h (Fig. 2). It is worth noting that CG-G4-1% absorbs 23 times as many as its dry mass at pH 7.4 within 15 min. CG-G4-1% and CG-G4-10% exhibit the highest and lowest equilibrium swelling ratio at pH 7.4, respectively. The different swelling ratios are attributed to 3-dimensional structure and porosity of the cryogel. In general, a loosely cross-linked network with high porosity allows more PBS to be absorbed into the swollen gel. For all the three cryogels, the swelling equilibrium at pH 1.2 is higher than that at pH 5.3 or pH 7.4. The effect of pH on swelling is especially pronounced for CG-G4-1%. The equilibrium swelling ratios of CG-G4-1% at pH 1.2, pH 5.3 and pH 7.4 are, respectively, 5130%, 2760%, and 2590% (Fig. 2a). The equilibrium swelling ratios of CG-G4-5% at pH 1.2, pH 5.3 and pH 7.4 are 1800%, 1200%, and 1100%, respectively (Fig. 2b). The equilibrium swelling ratios of CG-G4-10% at pH 1.2, pH 5.3 and pH 7.4 are 900%, 700%, and 550%, respectively (Fig. 2c). CG-G4-5% and CG-G4-10% can absorb 1.6 times medium at pH 1.2 as much as they can at pH 7.4.

**Figure 3:**
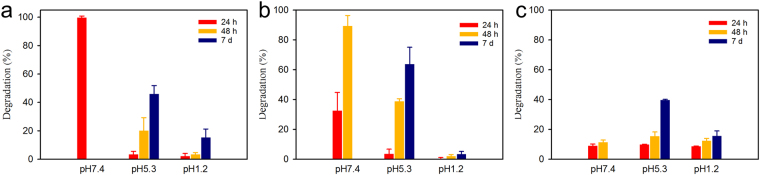
Degradation of dendrimer cryogels at 37 °C. (**a**) Effect of pH on the degradation of CG-G4-1%. (**b**) Effect of pH on the degradation of CG-G4-5%. (**c**) Effect of pH on the degradation of CG-G4-10%.

As shown in Fig. 3, the degradation rates of cryogel formulations at pH 7.4 from high to low are CG-G4-1%, CG-G4-5%, and CG-G4-10%. CG-G4-1% degrades completely within 24 h. CG-G4-5% shows 32% degradation in 24 h and 89% degradation in 48 h. Strikingly, there is no significant degradation of CG-G4-10% within 48 h. The degradation differences of the three cryogels at pH 7.4 are primarily caused by porosity and morphology. Dendrimer cryogels degrade much more slowly at pH 5.3 and pH 1.2 (Fig. 3a–c). Especially, the cryogels are most stable at pH 1.2. They had limited degradation even after 7-day incubation.

Dendrimer cryogels made of cationic dendrimer show pH-dependent swelling behavior. They have higher swelling ratios in acidic buffer than in neutral buffer. Polymeric networks possessing carboxylic acid or amine groups is capable of changing their swelling and disintegration behaviors in response to pH change. In general, the ionization of amines at low pH would make the polymer more hydrophilic, resulting in quicker swelling and degradation. Nonetheless, dendrimer cryogels degrade significantly slowly in acidic solutions. Especially, the degradation of all the three cryogels at pH 1.2 is almost suppressed within 7 days. We attribute the opposite degradation pattern of our dendrimer cryogels to the self-triggered degradation mechanism. Ester bonds form after the aza-Michael addition between PAMAM G4 and PEG-DA. The ammonolysis of ester bonds is triggered by the unreacted primary amine groups on dendrimer surface, thus leading to the degradation of the dendrimer cryogel network.

### Rheological and elastic properties

**Figure 4:**
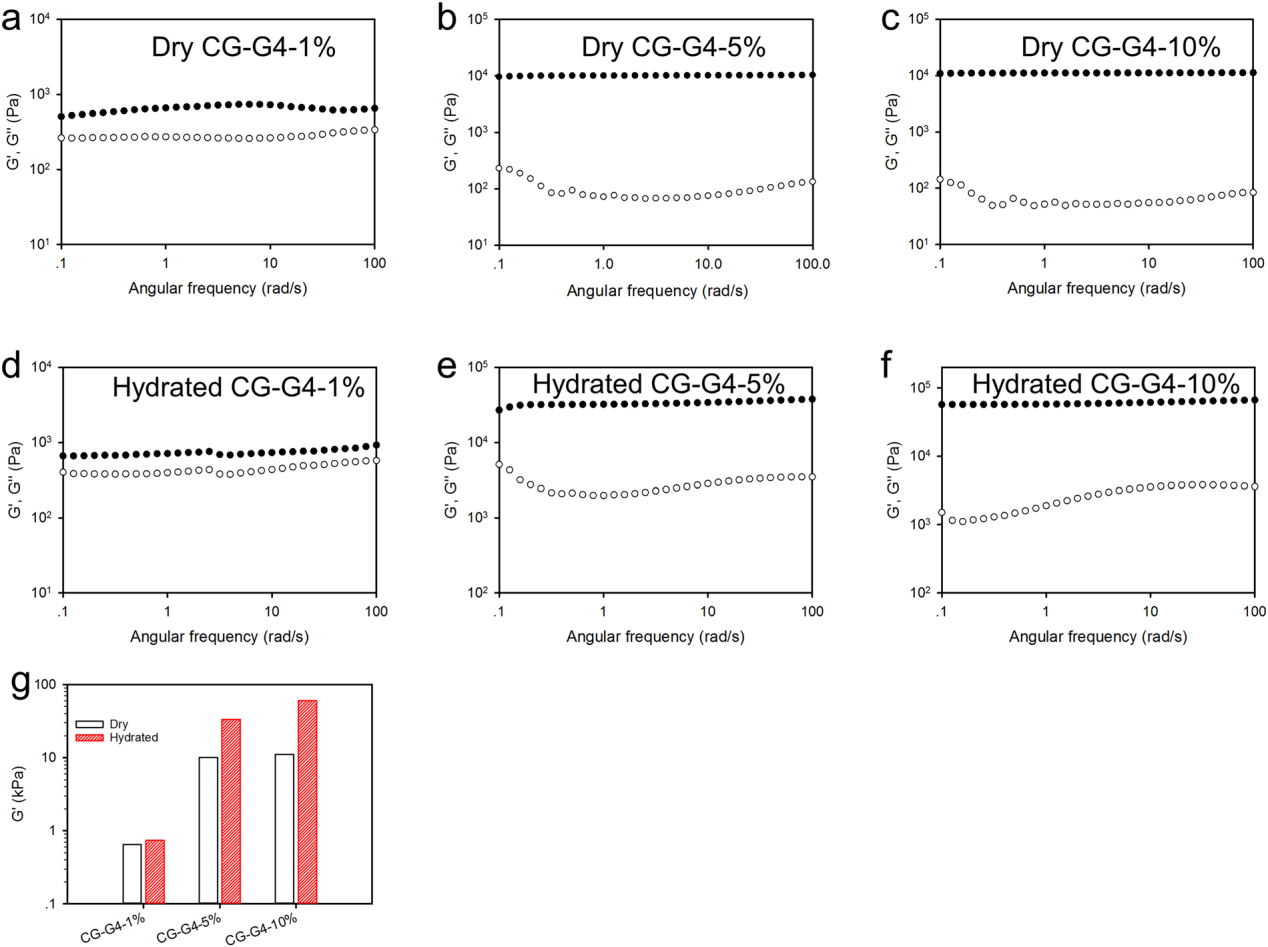
Oscillatory frequency sweep of CG-G4-1%, CG-G4-5% and CG-G4-10% in dry state (**a–c**) and hydrated state (**d–f**). (**g**) Storage modulus of the tested cryogels at 1 rad/s. ● Represents storage modulus (G′) and ○ represents loss modulus (G″).

An amplitude sweep was first carried out to make sure that the measurement was in the linear viscoelastic region (Figure S4). The frequency sweeps were then measured at a fixed strain of 1%. As shown in Fig. 4a–f, all the samples display typical viscoelastic behavior as their storage moduli (G′) are much higher than their loss moduli (G′′), and G′ is frequency-independent over the entire measured frequency range. It is interesting that the three hydrated cryogels have a higher modulus compared to their counterparts in the dry state, especially for CG-G4-5% and CG-G4-10% (Fig. 4g).

**Figure 5:**
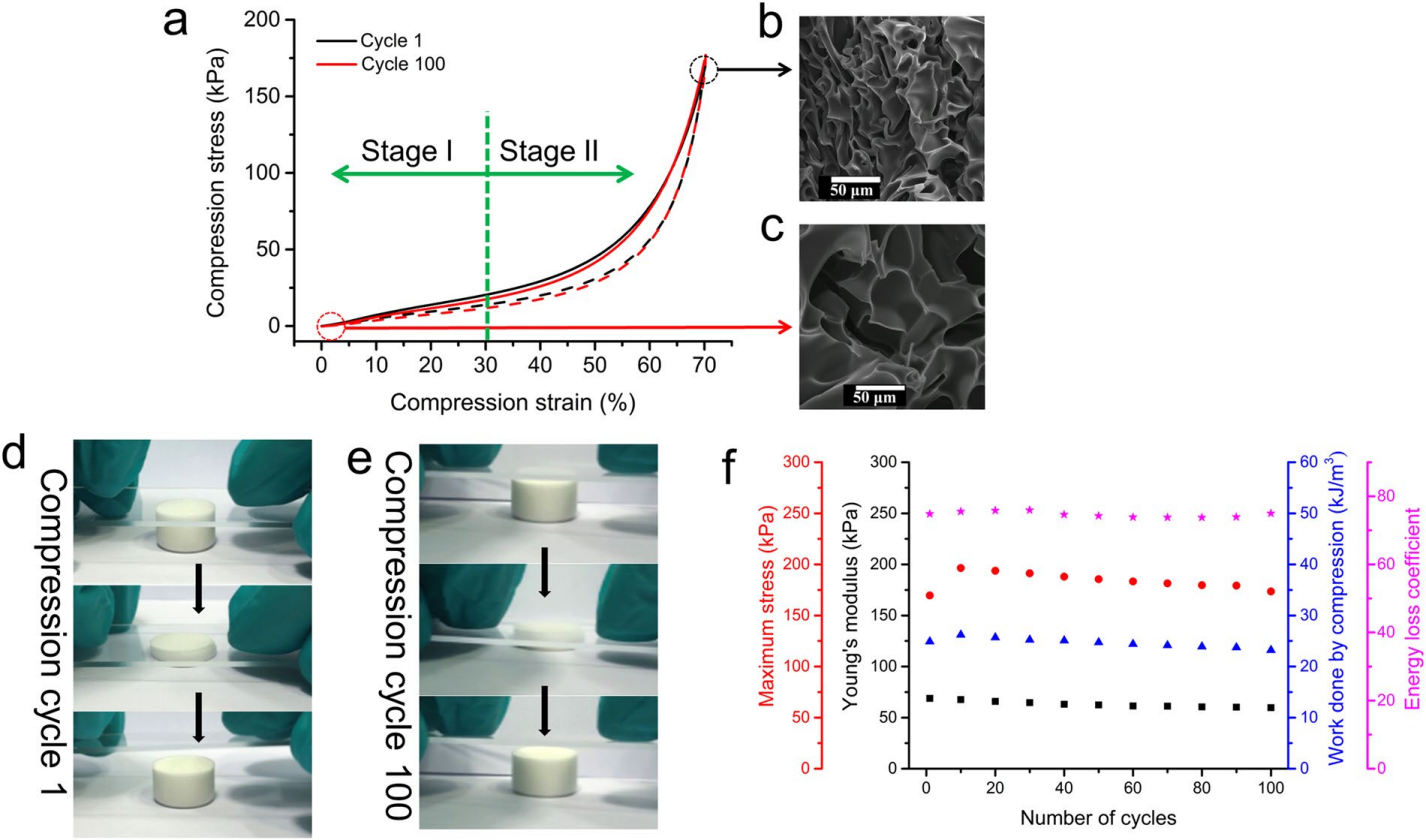
Cyclic compressive properties of CG-G4-5% at ambient temperature. (**a**) Compressive stress-strain curves of cycle 1 and cycle 100 of loading (solid lines) and unloading (dashed lines). (**b**) SEM image of the dendrimer cryogel under 70% strained compression in cycle 1. (**c**) SEM image of the dendrimer cryogel after recovery from 100 cycles of compression. (**d**) A set of real-time images to demonstrate the recovery of the dendrimer cryogel from one cycle of manual compression. (**e**) A set of real-time images to demonstrate the recovery of the dendrimer cryogel from 100 cycles of manual compression. (**f**) Young’s modulus (■), maximum stress (), work done by compression (), and energy loss coefficient () of the dendrimer cryogel experiencing different numbers of cycles of compression.

As shown in the compression stress-strain curve (Fig. 5a), there are two distinct stages of deformation observed. Dendrimer cryogel shows linear elasticity in stage I at low strains below 30%, which is attributed to the bending of the pore walls. Compression Young’s modulus (*E*_C_) is 72.5 ± 2.8 kPa. The work done by compression is used to compress the pores. The elastic deformation is recovered during the rebound phase (Figure S5). In contrast, at higher strains (30% to 70%) in stage II, stress increases abruptly. The work done by compression is responsible for the further buckling and densification of the pores. At 70% strain, dendrimer cryogel supports over 6400 times its weight without collapsing. This property is rarely seen in other polymeric elastomers^16,22,29,32–34^. Furthermore, the dendrimer cryogel exhibits excellent rebound performance and does not show significant stress relaxation under cyclic deformation at room temperature.

When compressed to at least 70% strain in the first cycle, the pores are closed and densificated (Fig. 5b). After unloading, it recovers quickly to its original shape. More impressively, this high resilience maintains even after many cycles of load-unload compression. As shown in Fig. 5c, the cryogel maintains open pores after recovery from a minimum of 100 cycles of compression. The cryogel recovers its initial shape and size instantly upon load removal following 100 times of manual compression (Fig. 5d,e and movie S1). The physical characteristics of the cryogel work done by compression, maximum stress, *E*_C_, energy loss coefficient are highly stable throughout the test (Fig. 5f).

We used the cryo-aza-Michael addition method to prepare and tested two aditional sets of crygels for comparision: chitosan-based cryogel CG-chitosan (non-dendrimer-based) and dendrimer cryogel using a lower generation dendrimer G1, i.e., CG-G1-5%. We tested the compression stress-strain properties on CG-G1-5% and CG-chitosan. CG-G1-5% exhibits a macroporous structure similar to that of CG-G4-5% (Figure S6a). Although CG-G1-5% has an *E*_C_ of 30.4 ± 0.1 kPa, which is lower than that of CG-G4-5%, its resilience is comparable to the resilence of CG-G4-5%. The compression stress-strain curve of cycle 100 is nearly identical to that of cycle 1 (Figure S6b). The work done by compression, maximum stress, *E*_C_, energy loss coefficient of the CG-G1-5% are highly stable throughout the test (Figure S6c). The CG-chitosan shows a porous structure (Figure S7a). It shows an higher *E*_C_ (333.6 kPa) than dendrimer cryogels. Different from dendrimer cryogels, CG-chitosan exhibits three stages of deformation (Figure S7b). The stress plateau in stage II is attributed to the elastic buckling of pore walls. However, CG-chitosan does not rebound (Figure S7b–d). The studies support the unique dendritic-linear cross-linked structure is primarily responsbile for the superior resilience property of dendrimer cryogels.

**Figure 6:**
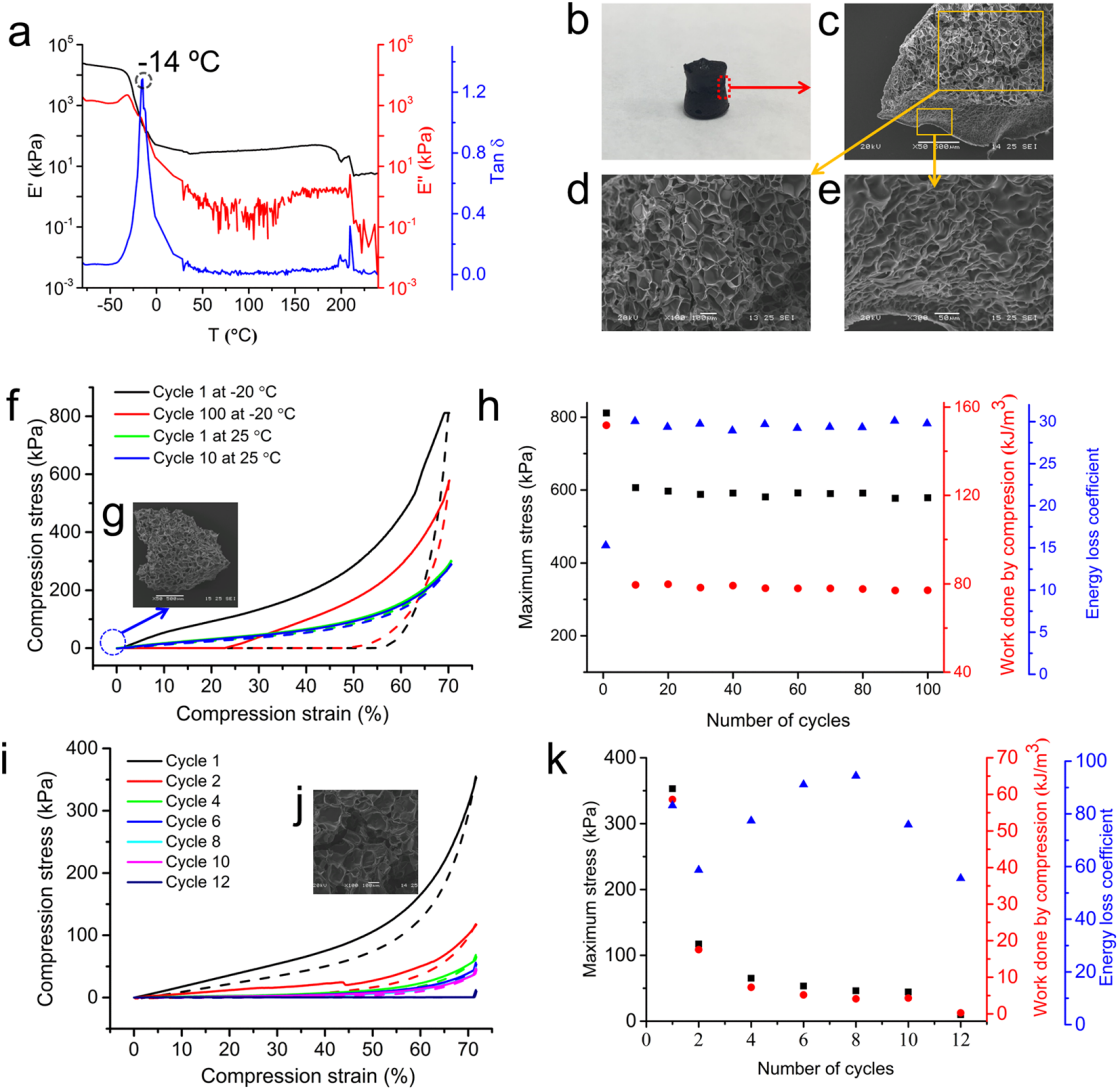
Cyclic compressive properties of CG-G4-5% at non-ambient temperatures. (**a**) Dynamic temperature ramp (DMA) from −80 °C to 240 °C showing the elastic modulus (black line), loss modulus (red line), and loss angle tangent (blue line). (**b**) The photo of carbonized dendrimer dendrimer cryogel after the DMA test. (**c–e**) The SEM images of carbonized dendrimer cryogel. Scale bars: 500 μm (**c**,**g**), 100 μm (**d**,**j**), and 50 μm (**e**). (**f**) Compressive stress-strain curves of cycle 1 and cycle 100 at −20 °C and the followed by additional 10 cycles at 25 °C of loading (solid lines) and unloading (dashed lines). (**g**) The SEM image of dendrimer cryogel after 100 cycles of compression at −20 °C and 10 cycles of compression at 25 °C. (**h**) The maximum stress during compression stress-strain cycle (black squares), work done by compression (red circles), and energy loss coefficient (blue triangles) of the dendrimer cryogel subjected to different numbers of cyclic compression −20 °C. (**i**) Compressive stress-strain curves of cycle 1, 2, 4, 6, 8, 10, 12 at 100 °C of loading (solid lines) and unloading (dashed lines). (**j**) The SEM image of dendrimer cryogel after 12 cycles of compression at 100 °C showing the cracked morphology. (**k**) The maximum stress during compression stress-strain cycle (■), work done by compression (), and energy loss coefficient () at different cycles during the 12 cycles of compression test at 100 °C.

We went on examining how temperature change affects the dynamic mechanical properties and the compression properties of dendrimer cryogel using dynamic mechanical analysis (DMA). The changes of storage modulus (E’), loss modulus (E”) and loss tangent (Tanδ) over a broad temperature range (−80 to 200 °C) are shown in Fig. 6a. The temperature corresponding to the peak value of tanδ, −14 °C, is identified as glass transition temperatures (*T*_g_). The dendrimer cryogel has high E’ values (>10^4^ kPa) at temperatures below *T*_g_, which is 2 orders of magnitude higher than at temperatures above *T*_g_. The dendrimer cryogel shows excellent elasticity below 190 °C. At the end of the DMA test, we noticed that the sample was carbonized with permanent necking deformation (Fig. 6b). The carbonized dendrimer cryogel maintains a macro-porous but anisotropic structure (Fig. 6c). Carbonized dendrimer cryogel is an entirely new type of material. There is no significant structural change in the direction perpendicular to necking (Fig. 6d); however, the densification of pores is seen in the direction of necking (Fig. 6e). Further investigation on the carbonized dendrimer cryogelis warranted but beyond of the scope of this paper. The rebound performance of the elastomer at non-ambient temperatures, i.e., −20 and 100 °C, was investigated. The elastomer at −20 °C is much stiffer than at the ambient temperature as its peak stress increases nearly 5 folds to 811 kPa (Fig. 6f). Also, the hysteresis loop is larger. The elastomer recovers its high elasticity quickly upon cyclic compression. The energy loss coefficient is 15.3% in cycle 1 (Fig. 6h). It goes higher as the elastomer receives 10 cycles of compression and remains constant at 30% even under a higher number of cyclic compression. The initial work done by compression and maximum stress quickly drop to a stable level after cyclic compression is applied 10 times or more. The dendrimer cryogel shows suboptimal elasticity and resilience property at −20 °C compared to itself at ambient temperature. Nonetheless, its overall rebound performance at −20 °C is superior to what most elastomers exhibit at ambient temperature. It was worth noting that the stress-strain behaviors of the elastomer (green and blue lines in Fig. 6f) almost recover to its initial state upon extra cycles of load-unload compression when the temperature is increased immediately to 25 °C. There is no significant difference in hysteresis loop between cycle 1 and cycle 10. The morphological structure of the dendrimer cryogel at the end of the experiment (Fig. 6g) looks similar to what is observed in the untreated sample. In contrast, the dendrimer cryogel quickly loses its elastomeric characteristics at 100 °C (Fig. 6i and k). The sample was crushed after 12 cyclic compressions accomplished with loss of porous structure (Fig. 6j).

### Fast recovery of superelasticity after storage

**Figure 7:**
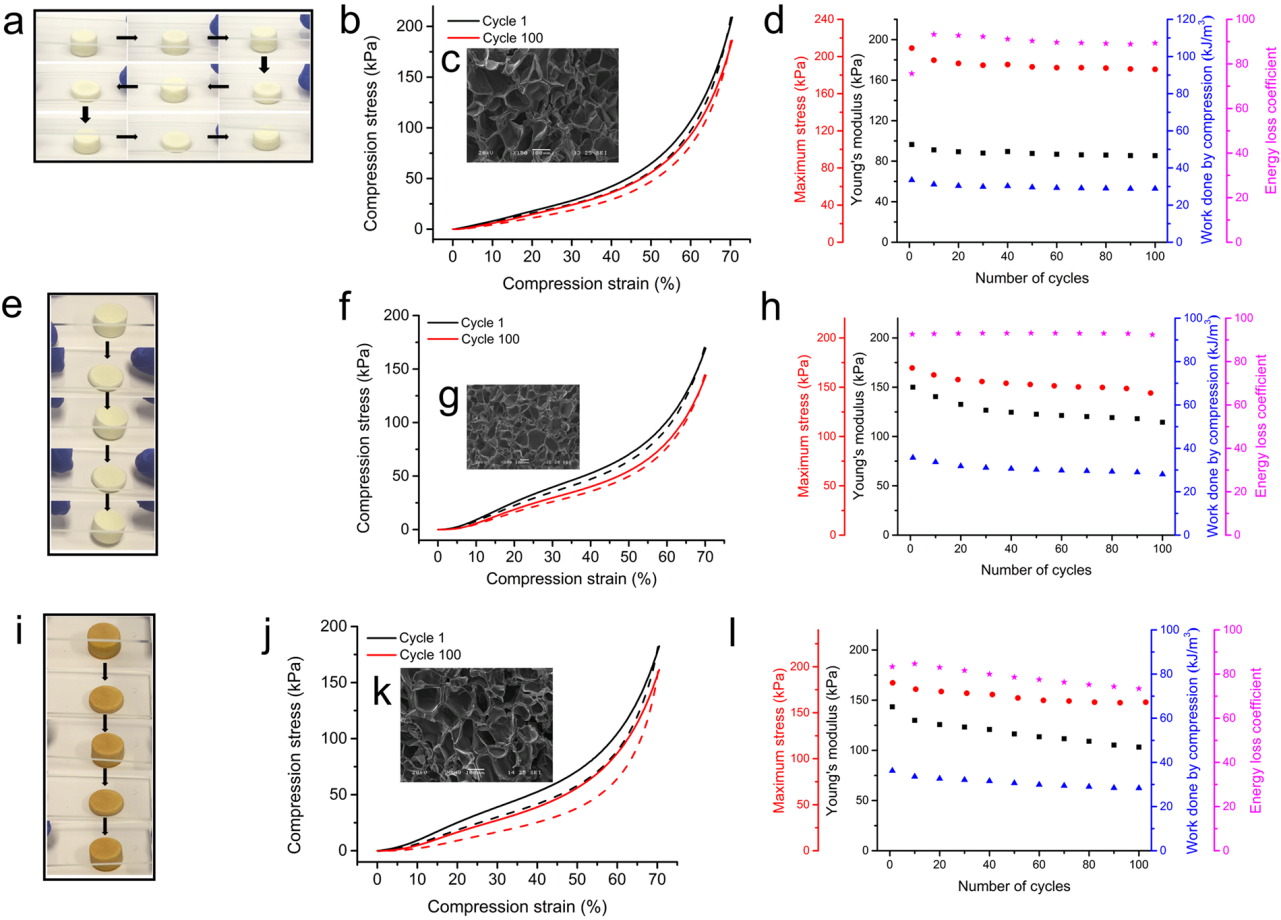
The ultra-fast recovery of high resilience of CG-G4-5% after storage at high and low temperatures. (**a**) A set of real-time images of a dendrimer cryogel sample after 24 h storage at −80 °C showing the recovering process at cycle 1, 2, 3, and cycle 4. (**b**,**f**,**j**) Compressive stress-strain curves of cycle 1 and cycle 100 of loading (solid lines) and unloading (dashed lines) at 25 °C after storage at −80, −20, and 100 °C for 24 h. (**c**) The SEM images of dendrimer cryogel after storage at −80 °C for 24 h and then being compressed 100 cycles. (**d**,**h**,**l**) Young’s modulus (■), maximum stress (), work done by compression (), and energy loss coefficient () at different cycles during the 100 cycles of compression test after storage at −80, −20, and 100 °C for 24 h. (**e**) A set of real-time images of a dendrimer cryogel sample after 24 h storage at −20 °C showing the recovering process at cycle 1 and 2. (**g**) The SEM images of dendrimer cryogel after storage at −20 °C for 24 h and then being compressed 100 cycles. (**i**) A set of real-time images of a dendrimer cryogel sample after 24 h storage at 100 °C showing the recovering process at cycle 1 and 2. (**k**) The SEM images of dendrimer cryogel after storage at 100 °C for 24 h and then being compressed 100 cycles. Scale bars: 100 μm (**c**,**g**,**k**).

Encouraged by the observation of the rapid recovery from 100 cycles of compression at −20 °C, we studied the performance after storage at −80, −20, and 100 °C for 24 h, respectively. The studies show that the dendrimer cryogel rapidly restores high resilience property after storage at different temperatures. One hundred relatively constant manual compressions were applied to illustrate the resilience properties of the stored elastomers recovering at ambient temperature. After 24 h storage at −80 °C, the sample can be only compressed to approximately 5% strain in cycle 1. Approximately 50% strain and 60% strain could be compressed in cycle 2 and cycle 3, respectively. It recovered its high elasticity in cycle 4 with no less than 70% strain (Fig. 7a, movie S2). Similarly, the dendrimer cryogel restores its excellent elasticity immediately at ambient temperature after being stored at either −20 °C (Fig. 7e, movie S3) or 100 °C (Fig. 7i, movie S4) although the color changed to yellow after storage at 100 °C for 24 h. Quantitative cyclic compression tests confirm that the dendrimer cryogels retain the superelasticity at ambient temperature (Fig. 7b,f,j). No significant decreases in work done by compression, maximum stress, Young’s modulus, and energy loss coefficient are observed after 100 cycles of compression (Fig. 7d,h,l). The porous structures of the samples stay unchanged after storage and subsequent cyclic compressions (Fig. 7c,g,k).

**Figure 8:**
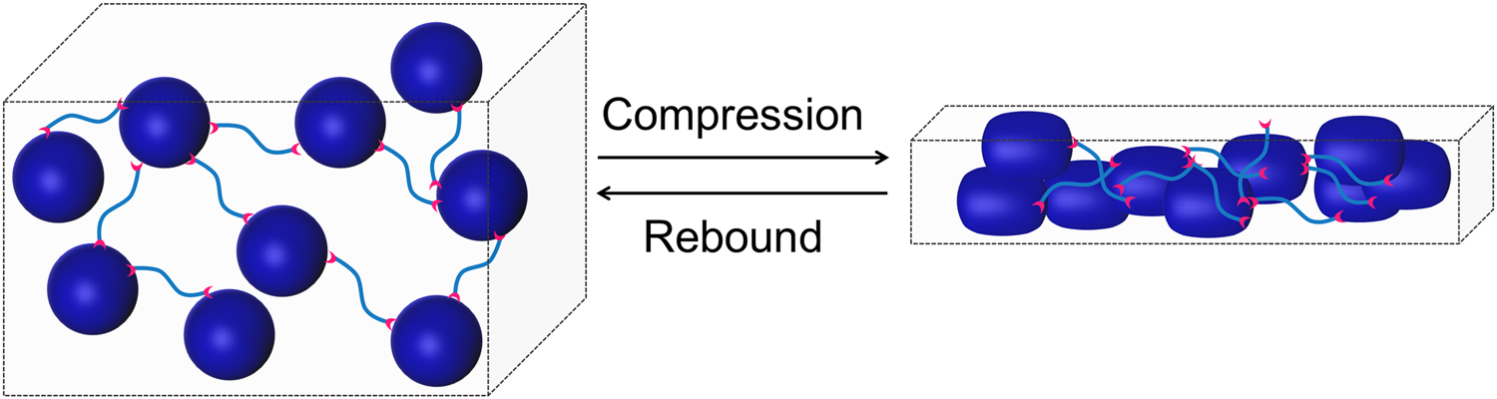
A macroporous cross-linked nanoelastomer-linear polymer network is attributed to the superelasticity and super resilience of dendrimer cryogels prepared via the cryo-aza-Michael addition of hyperbranched polyamidoamine dendrimer and linear PEG.

From the molecular perspective, single dendrimer molecule is elastic^35^. The cryopolymerization organizes the randomly dispersed dendritic nanoelastomers into a more relatively organized cross-linked network knotted with linear PEG chains. The subsequent freeze-drying endows it with a macroporous structure upon the removal of ice crystals. The bulky macroporous structure made of cross-linked nano-elastomer–linear polymer collectively contribute to a hierarchical elasticity across different lengths of scale (Fig. 8). It is well accepted that cross-linking can affect the flexibility of polymer chains and hence *T*_g_. When the degree of cross-linking is high, the conformational change of polymer segments would be difficult, thus cuasing *T*_g_ to increase significantly or disappear. Due to the 3-D spherical nanostructure of the dendrimer, the dendritic-linear network greatly reduces the effect of cross-linking on the movement of polymer segments. This unique hierarchical elasticity coupled with unrestricted segmental motion between cross-links accounts for a very low *T*_g_ of the resulting dendrimer cryogel. We are currently exploring dendrimer cryogels for drug delivery.

In conclusion, the aza-Michael addition-based cryopolymerization of super atomistic dendritic building blocks and flexible linear PEG chains leads to the development of dendrimer cryogels with superelasticity, high resilience, and pH-dependent swelling and degradation. The dendrimer cryogels have ultralightweight, an extremely high recovery rate after storage at various temperatures. Dendrimer cryogels are a new class of materials for drug delivery and tissue engineering applications.

## Methods

### Materials

EDA-core PAMAM dendrimer G4.0 (G4) and G1.0 (G1) were purchased from Dendritech (Midland, MI). Polyethylene glycol diacrylate (PEG DA, *M*_n_ = 575 g/mol) and chitosan (*M*_w_ = 50 000 −190 000 Da) were purchased from Sigma-Aldrich.

### Fabrication of Dendrimer Cryogels

PAMAM G4 at different concentrations (1, 5, and 10 w%) were dissolved in deionized water equilibrated at 25 °C. The prepared PAMAM G4.0 aqueous solution and PEGDA were precooled to 4 °C before use. PEGDA was added to the dendrimer aqueous solution (the molar ratio of amine/acrylate = 1/1), and the mixture was vortexed for 30 s at 25 °C. The mixture was then transferred to a 5 ml Teflon beaker (20 mm OD, 17 mm ID, 26 mm H) and kept at −20 °C to allow for the cryopolymerization. After 17 h cryopolymerization, the resulting dendrimer cryogels (CG-G4-1%, CG-G4-5%, and CG-G4-10%) were obtained after freeze-drying. CG-G1-5% was prepared following the same procedure as CG-G4-5%. Chitosan cryogel (CG-chitosan) was prepared mixing 2 mL of saturated chitosan solution (approximately 17 mg chitosan) in pH 2 buffer (Na_2_HPO_4_-citric acid) with 28 mg of PEG DA. The mixture was vortexed for 30 s at 25 °C, transferred to a 5 ml Teflon beaker and kept at −20 °C to allow for cryopolymerization. After 17 h cryopolymerization, the resulting CG-chitosan was obtained after freeze-drying.

### Scanning Electron Microscopy (SEM)

The morphological features of the cryogels were examined under a scanning electron microscope JEOL LV-5610 or a field emission SEM microscope (Hitachi FE-SEM Su-70). Lyophilized cryogels mounted on carbon tapes were coated with platinum for 90 seconds using an ion sputter. To image compressed dendrimer cryogel samples with SEM, the samples were placed in a clamp to maintain a strain of approximately 70% as determined by using a caliper. SEM images were analyzed using ImageJ2 software analysis to determine the pore size and wall thickness of the dendrimer cryogel. The analysis was performed on at least three samples and at least 20 measurements per sample.

### Porosity Measurements

The porosity of the cryogel was estimated by using the hexane uptake method. The freeze-dried cryogels were first weighed and then immersed in hexane for 1 h, excess hexane was removed, and the samples were weighted. Porosity was calculated using the following equation:

$$P \% =\,\frac{\frac{{m}_{{\rm{s}}}-{m}_{{\rm{d}}}}{{\rho }_{{\rm{Hex}}}}}{\frac{{m}_{{\rm{d}}}}{{\rho }_{{\rm{CG}}}}}\times 100$$

where *m*_d_ and *m*_s_ are the weights of the cryogels before and after saturated hexane uptake, *ρ*_Hex_ and *ρ*_CG_ are the density of hexane and cryogels. The densities of cryogels were calculated by the ratio of mass to volume of the cylindrical cryogels with defined dimensions.

### Swelling Studies

Water absorption kinetics of cryogels (CG-G4-1%, CG-G4-5%, and CG-G4-10%) was determined. Each lyophilized cryogel was immersed and incubated in 1 mL of PBS (pH = 7.4) at 37 °C. The supernatant was gently sucked out at different time intervals, and the swollen hydrogel sample was weighed. The measurement period was up to 12 h to reach the maximum absorption. The swelling ratio (%) = (*W*_t_ − *W*_0_)/*W*_0_ × 100, where *W*_t_ represents the mass of the swollen sample and *W*_0_ represents the initial mass of the dry sample. The swelling kinetics of all the three cryogel in pH 5.3 PBS and pH 1.2 PBS were also studied following the same procedure as above.

### Degradation Studies

Each lyophilized cryogel was weighed as *m*_0_ and then saturated with PBS (pH = 7.4, 5.3, 1.2) at 37 °C. After the saturation, 2 mL fresh PBS (pH = 7.4, 5.3, 1.2) was added and incubated at 37 °C for 24 h, 48 h and 7 days, respectively. At different time intervals PBS was gently sucked out and samples were freeze-dried and re-weighed as *m*_de_. The degree of degradation (%) = (*m*_0_ − *m*_de_)/*m*_0_ × 100.

### Fourier Transform Infrared Spectroscopy (FTIR)

FTIR spectra of PAMAM dendrimer G4, PEG DA, CG-G4-1%, CG-G4-5%, and CG-G4-10% in dry state were recorded on Nicolet iS5 (ThermoFisher Scientific) equipped with iD7 ATR.

### Rheological Measurements

The rheological test was performed on samples (20 mm diameter disks). The test was conducted on both dry and hydrated samples on a temperature controlled plate of a Discovery Hybrid Rheometer (HR-3, TA instruments). Hydrated samples were immersed in 1 mL of PBS for 1 h before blot drying. Each set of samples on the plate at 37 °C was subjected to compression and shear stress by a 20 mm diameter parallel plate. An amplitude sweep was performed to confirm that all the measurements were conducted within the linear viscoelastic region. Oscillatory frequency sweeps were then carried out with a constant strain of 1% in the frequency region of 0.1–100 rad/s.

### Compression Testing

Cylindrical dendrimer cryogels with defined dimensions were subjected to compression test on a TA Instruments RSA-III dynamic mechanical analyzer (DMA). The samples (~12 mm in diameter and 8 mm in height) for compression tests were compressed up to 70% of their original length at the rate of 0.01 mm/s. The compression modulus was calculated from the slope of the linear section of the stress-strain curve. For cyclical compression test at −20 °C, 25 °C, and 100 °C, the rate of 0.2 mm/s was adopted. The strain of cyclical compression was fixed to 70%. The specimens (~7 mm in diameter and 9 mm in height) for dynamic temperature ramp tests were carried out under a nitrogen atmosphere from −80 °C to 240 °C at a temperature ramp of 5 °C/min. The frequency and strain were set at 1 Hz and 1%, respectively. One hundred relatively constant manual compressions were applied to CG-G4-5% after 24 h storage at −80, −20, and 100 °C, respectively.

## Electronic supplementary material

Supporting Information

Movie S1

Movie S2

Movie S3

Movie S4
