# Supplementary material for: Superelastic and pH-Responsive Degradable Dendrimer Cryogels Prepared by Cryo-aza-Michael Addition Reaction
Source: Sci Rep. 2018 May 8;8:7155. doi: 10.1038/s41598-018-25456-y (PMC5940921; doi:10.1038/s41598-018-25456-y)
Supplement: Supplementary file 1 — Supporting Information [file 41598_2018_25456_MOESM1_ESM.docx]

Supporting Information

Superelastic and pH-Responsive Degradable Dendrimer Cryogels Prepared by Cryo-aza-Michael Addition Reaction

Juan Wang^1^, Hu Yang^1,2,3,^*

^1^Department of Chemical and Life Science Engineering, Virginia Commonwealth University, Richmond, Virginia 23219, United States

^2^Department of Pharmaceutics, Virginia Commonwealth University, Richmond, Virginia 23298, United States

^3^Massey Cancer Center, Virginia Commonwealth University, Richmond, Virginia 23298, United States

* hyang2@vcu.edu


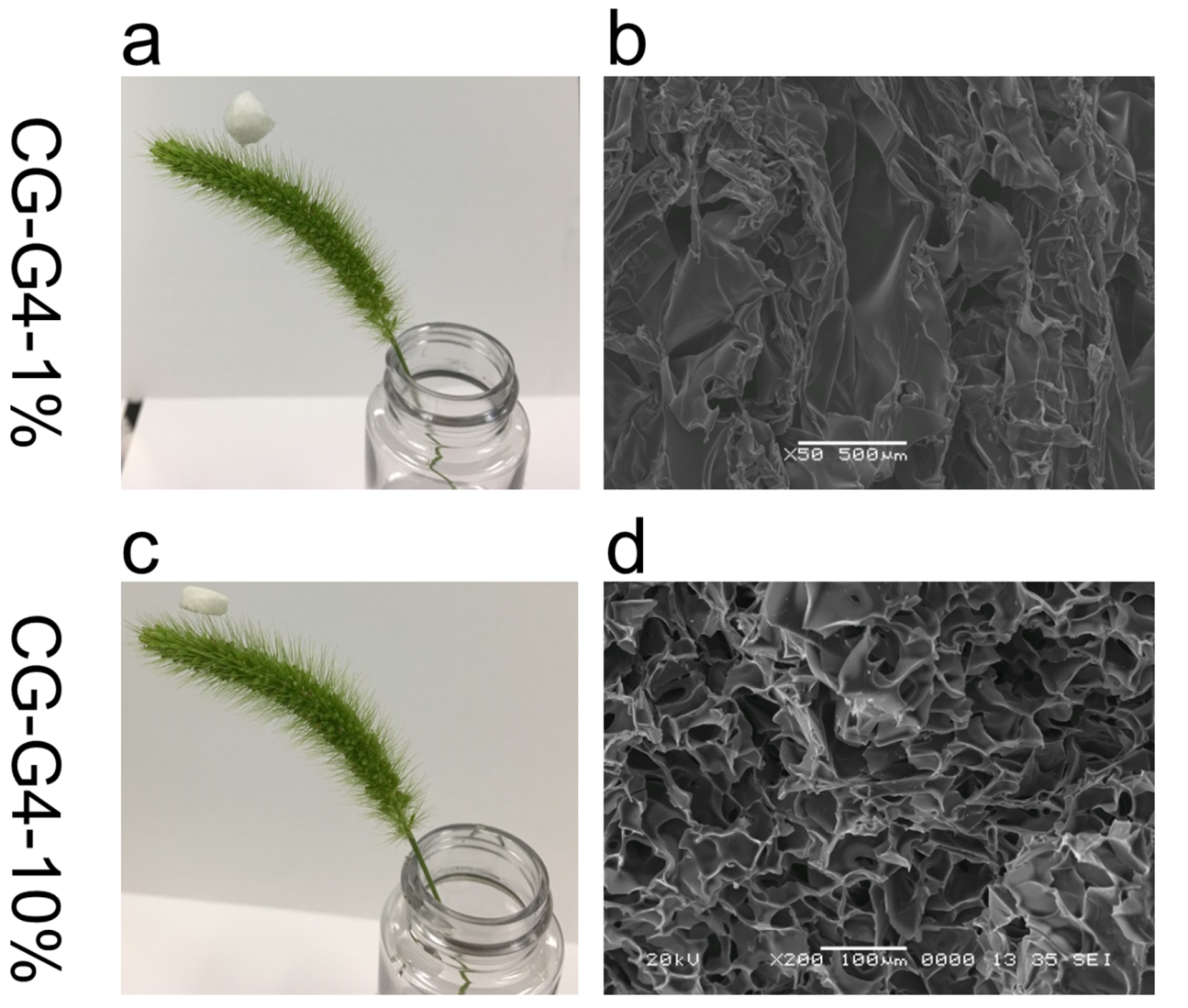


Figure S1. A Setaria viridis supports the dendrimer cryogels CG-G4-1% (a) and CG-G4-10% (c). SEM images of the dendrimer cryogels CG-G4-1% (b) and CG-G4-10% (d).


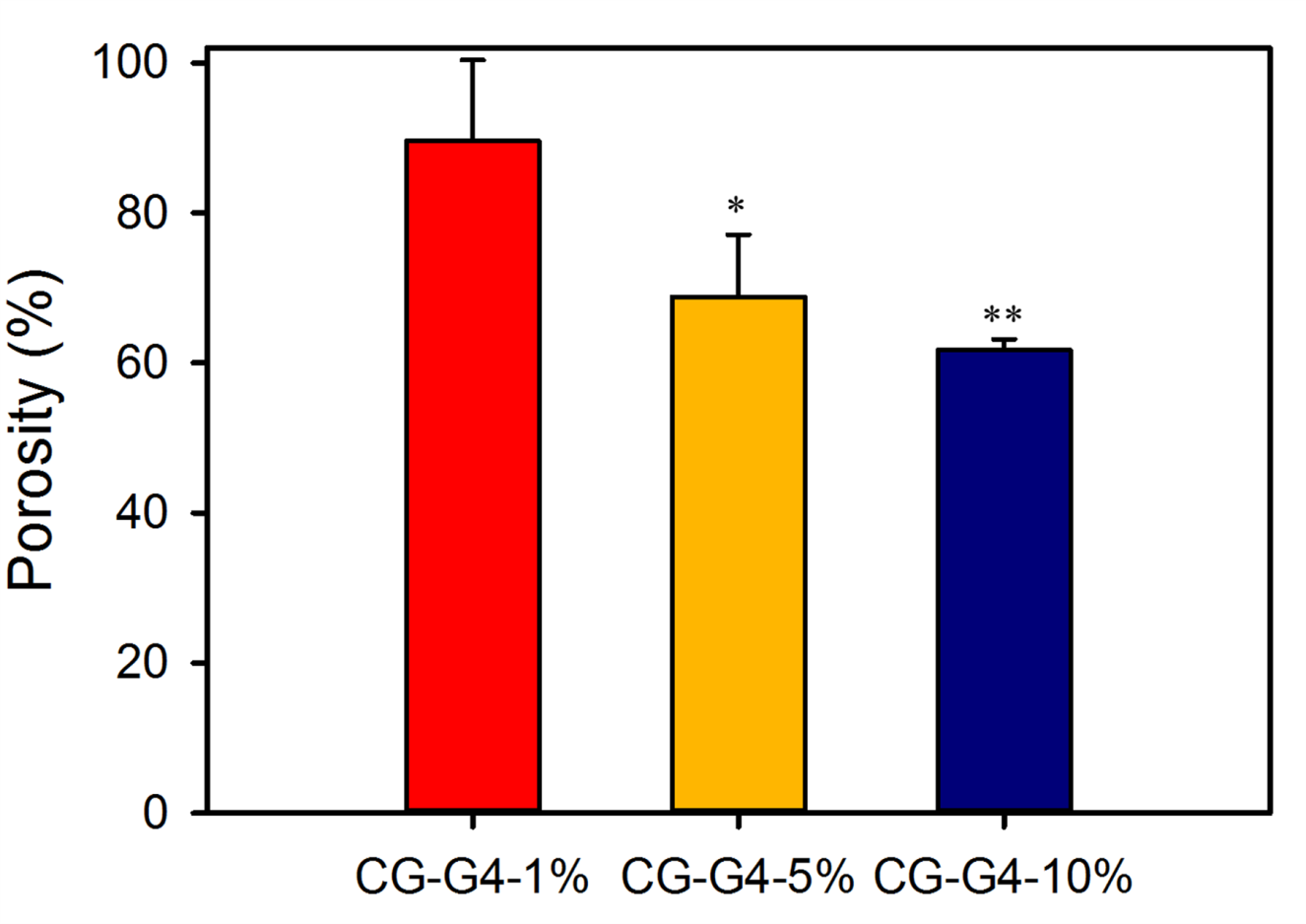


**Figure S2.** Porosities of CG-G4-1%, CG-G4-5% and CG-G4-10%. * P < 0.05, ** P < 0.01.


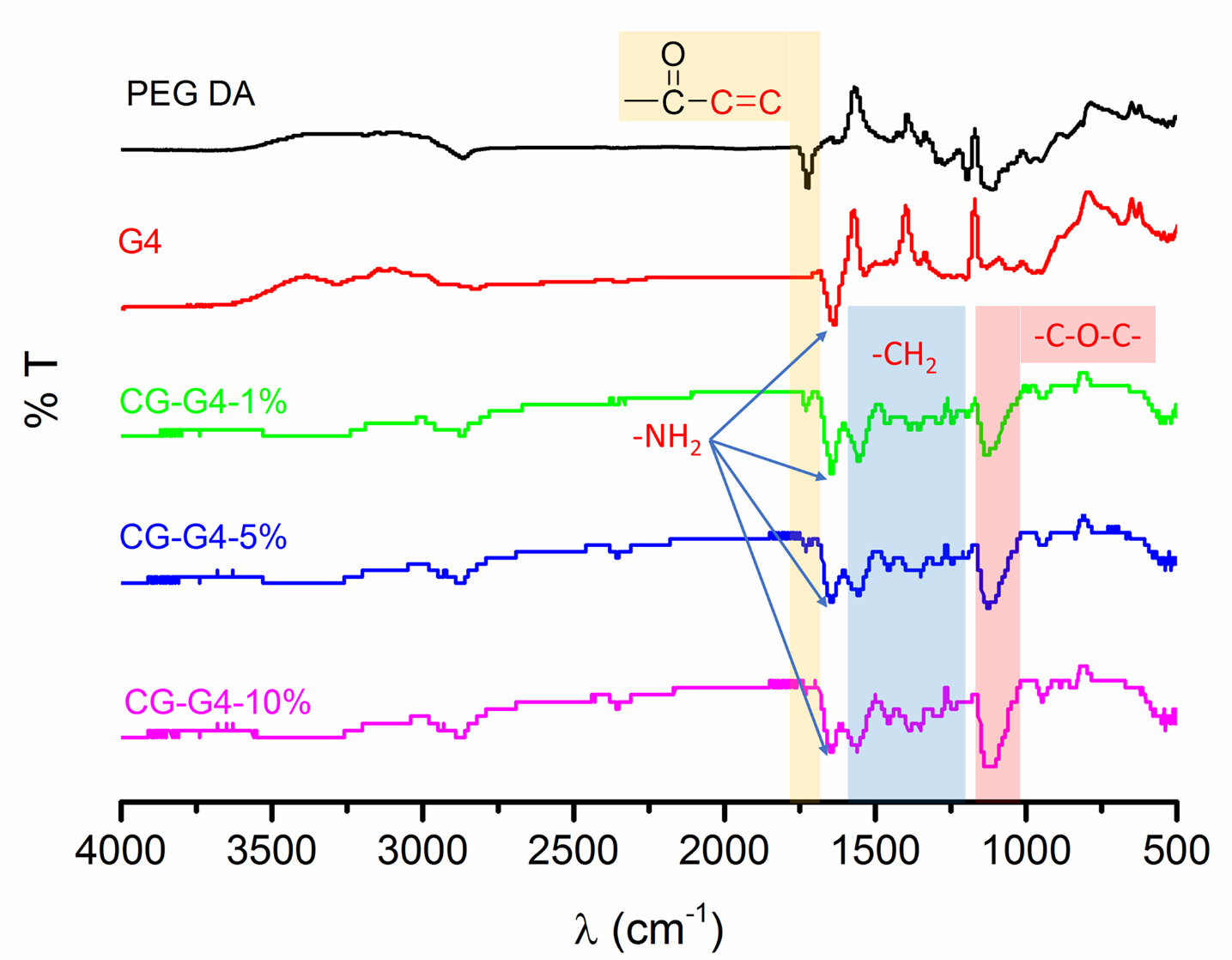


Figure S3. FTIR spectra of PAMAM dendrimer G4, PEG DA, CG-G4-1%, CG-G4-5%, and CG-G4-10%.


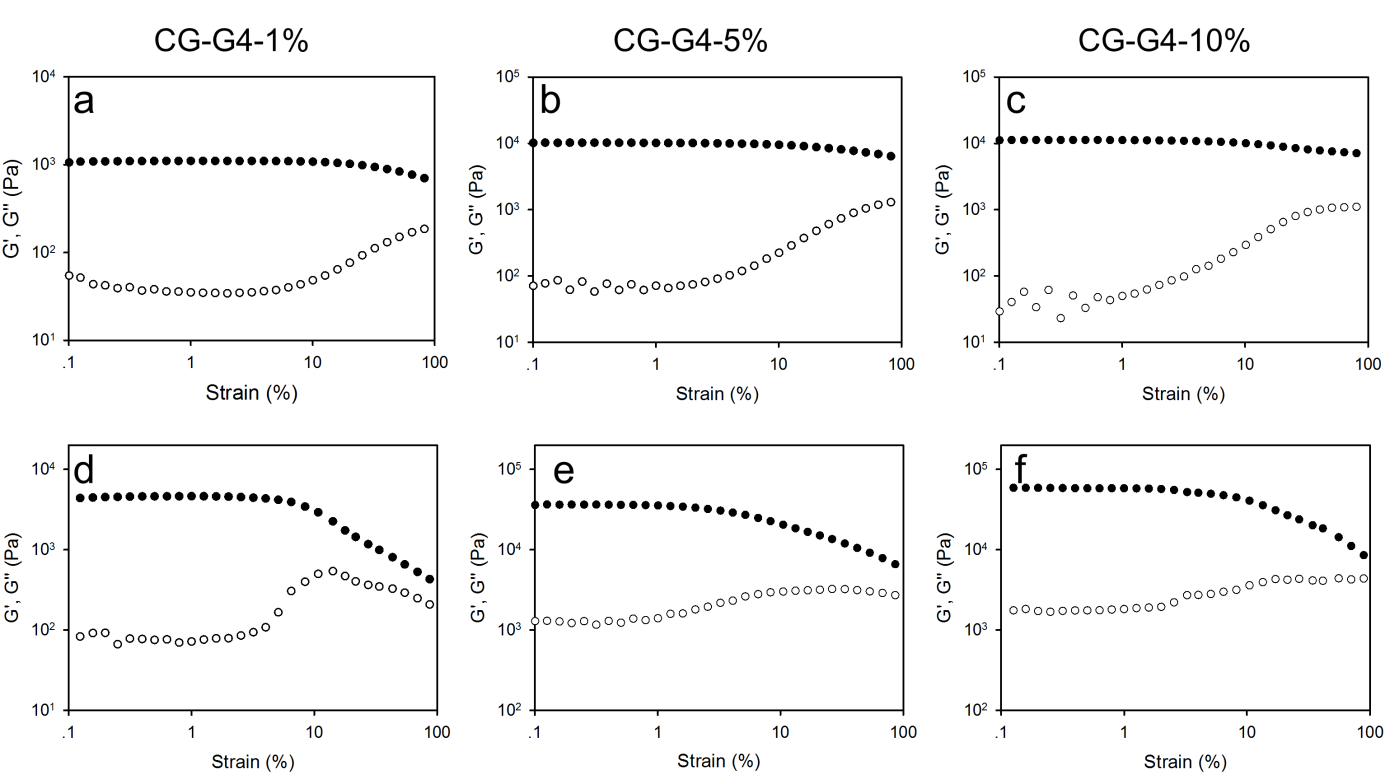


Figure S4. Oscillatory amplitude sweep of CG-G4-1%, CG-G4-5% and CG-G4-10% in dry state (a-c) and hydrated state (d-e). ● represents storage modulus (Gʹ), and ○ represents loss modulus (Gʹʹ) of cryogels.


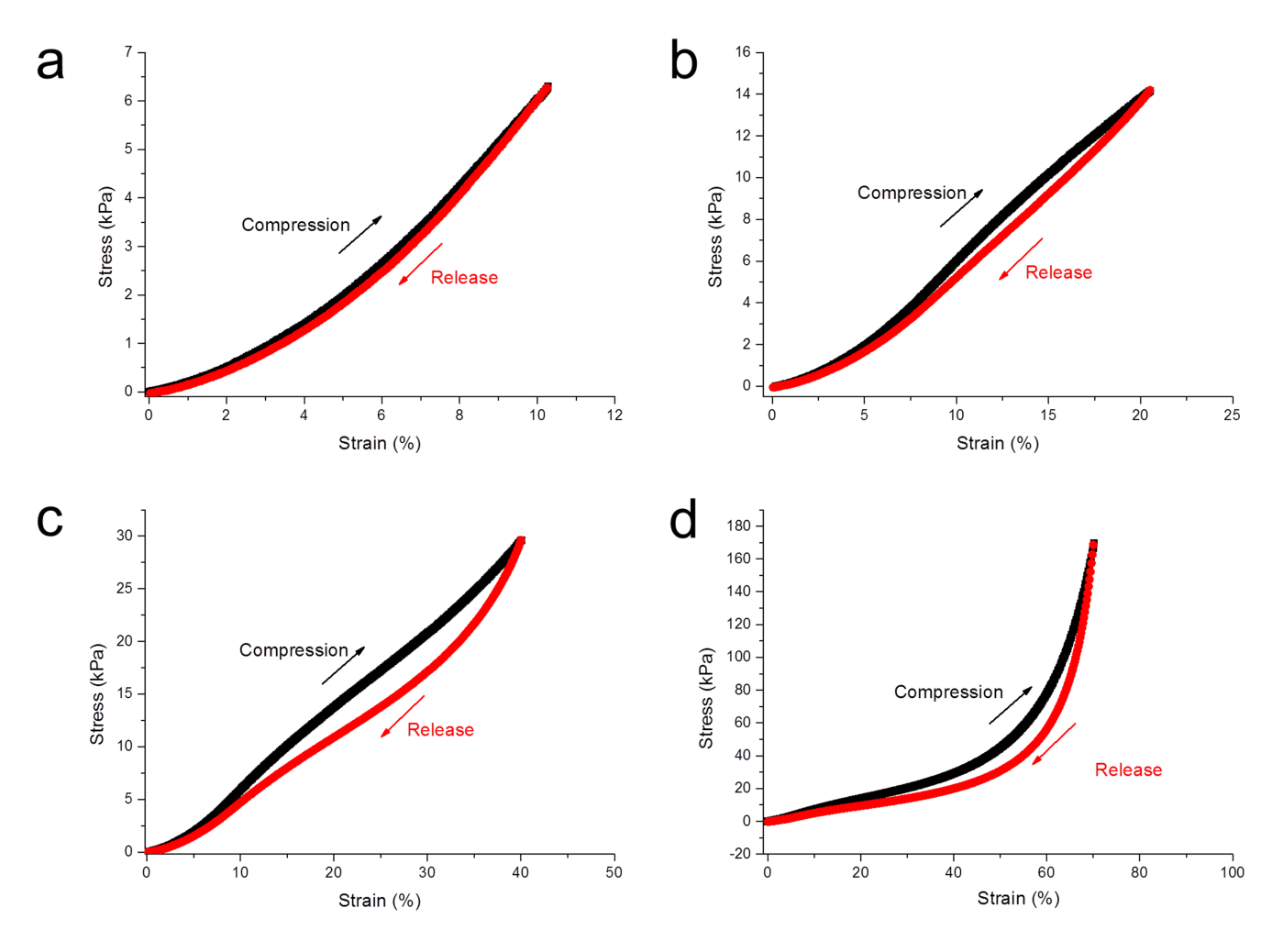


**Figure S5.** Compressive stress-strain curves of loading (black) and unloading (red) at different max. strains at 25 °C for CG-G4-5%.


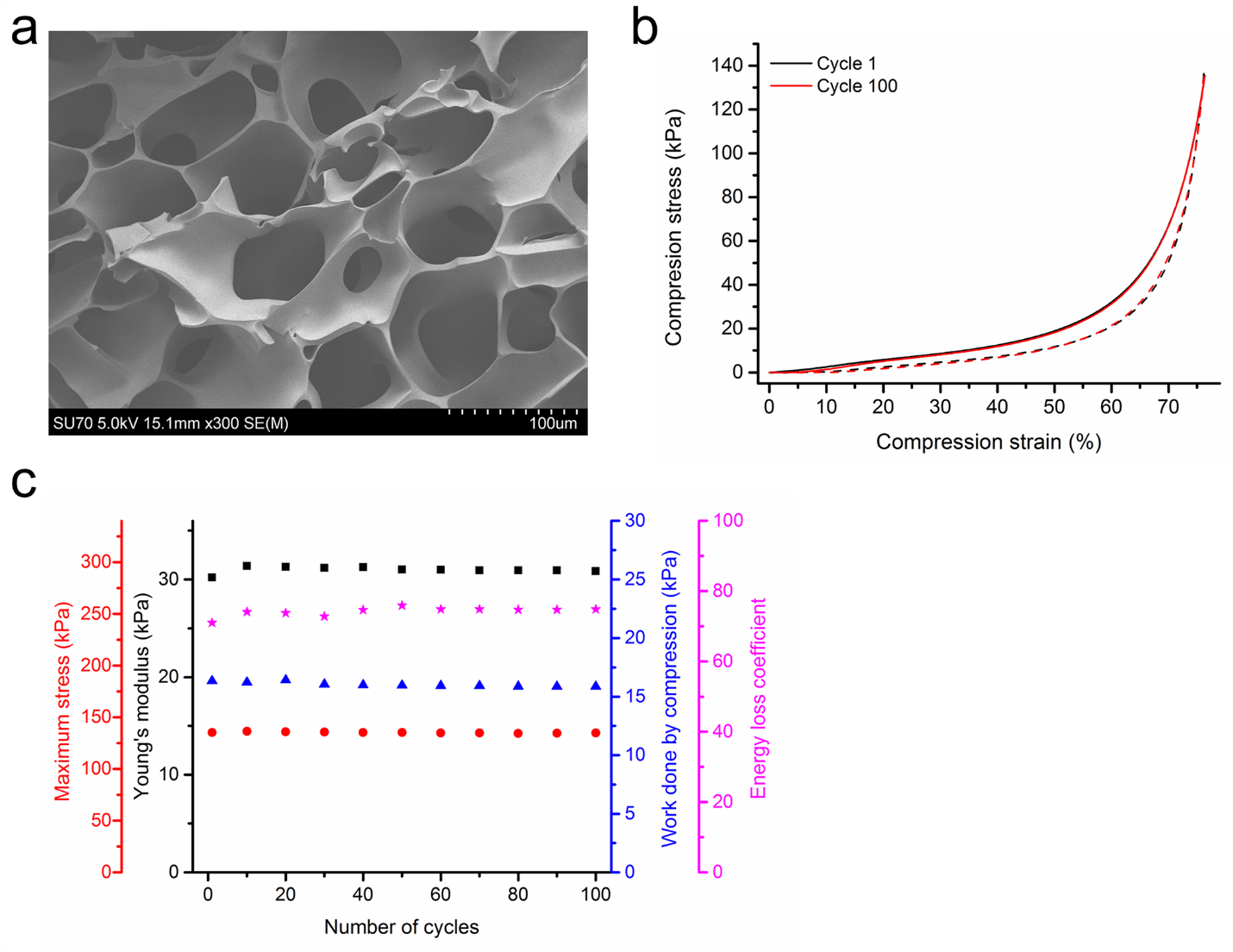


**Figure S6.** (**a**) SEM image of CG-G1-5%. (**b**) Compressive stress-strain curves of CG-G1-5% of cycle 1 and cycle 100 of loading (solid lines) and unloading (dashed lines). (**c**) Young’s modulus (⏹), maximum stress (⚫), work done by compression (▲), and energy loss coefficient (🟊) of the dendrimer cryogel experiencing different numbers of cycles of compression.


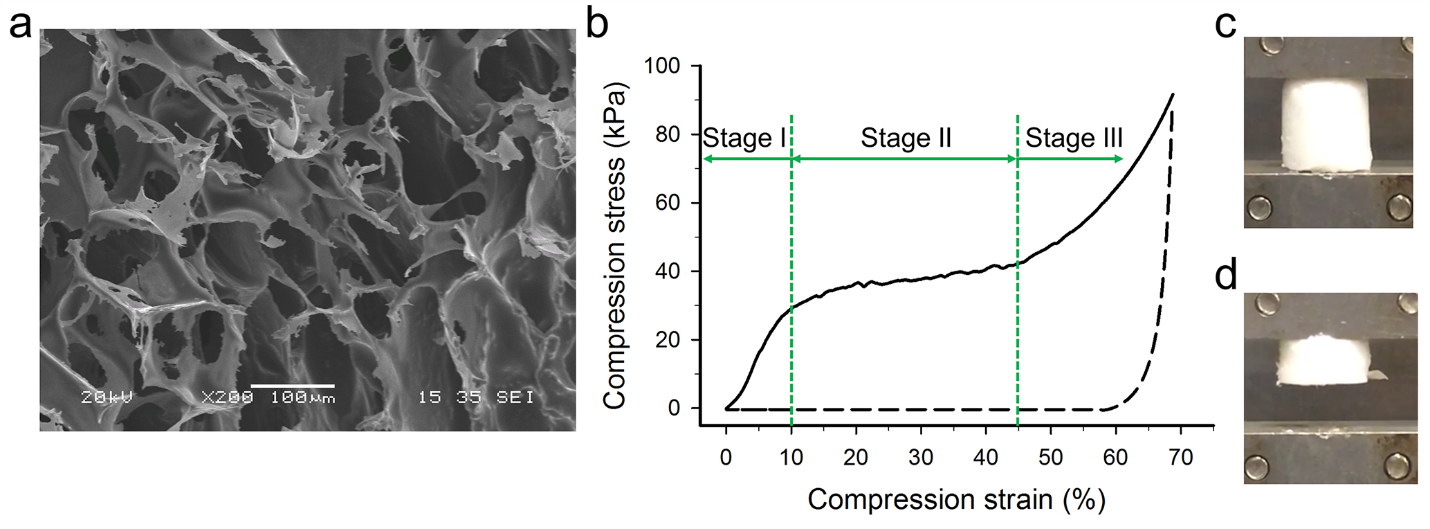


**Figure S7.** (**a**) SEM image of CG-chitosan. (**b**) Compressive stress-strain curves of CG-chitosan of loading (solid lines) and unloading (dashed lines). (c) A picture of CG-chitosan prior to compression force being applied. (d) A picture of CG-chitosan after the compression force was removed .

**Movie Legends**

**Movie S1.** 100 cycles of manual compression on CG-G4-5% at ambient temperature.

**Movie S2.** 100 cycles of manual compression on CG-G4-5% at ambient temperature after 24 h-storage at -80 °C.

**Movie S3.** 100 cycles of manual compression on CG-G4-5% at ambient temperature after 24 h-storage at -20 °C.

**Movie S4.** 100 cycles of manual compression on CG-G4-5% at ambient temperature after 24 h-storage at 100 °C.
